# “I have nine specialists. They need to swap notes!” Australian patients’ perspectives of medication‐related problems following discharge from hospital

**DOI:** 10.1111/hex.12556

**Published:** 2017-03-17

**Authors:** Daniela Eassey, Andrew J. McLachlan, Jo‐anne Brien, Ines Krass, Lorraine Smith

**Affiliations:** ^1^ Faculty of Pharmacy Camperdown The University of Sydney Sydney NSW Australia; ^2^ Centre for Education and Research on Ageing Concord Repatriation General Hospital Concord NSW Australia

## Abstract

**Background:**

Research has shown that patients are most susceptible to medication‐related problems (MRPs) when transitioning from hospital to home. Currently, the literature in this area focuses on interventions, which are mainly orientated around the perspective of the health‐care professional and do not take into account patient perspectives and experiences.

**Objective:**

To capture the experiences and perceptions of Australian patients regarding MRPs following discharge from hospital.

**Design:**

A cross‐sectional study was conducted using a questionnaire collecting quantitative and qualitative data. Thematic analysis was conducted of the qualitative data.

**Setting and participants:**

Survey participants were recruited through The Digital Edge, an online market research company. Five hundred and six participants completed the survey.

**Results:**

A total of 174 participants self‐reported MRPs. Two concepts and seven subthemes emerged from the analysis. The first concept was types of MRPs and patient experiences. Three themes were identified: unwanted effects from medicines, confusion about medicines and unrecognized medicines. The second concept was patient engagement in medication management, of which four themes emerged: informing patients, patient engagement, communication amongst health‐care professionals and conflicting advice.

**Discussion and conclusion:**

This study provides an important insight into patients’ experiences and perceptions of MRPs following discharge from hospital. Future direction for practice and research should look into implementing patient‐centred care at the time of hospital discharge to ensure the provision of clear and consistent information, and developing ways to support and empower patients to ensure a smooth transition post‐discharge from hospital.

## 1 INTRODUCTION

A growing body of evidence has firmly established that patients are most susceptible to experiencing adverse events during the transition from hospital to home.^1^, ^2^ It is estimated that approximately 20% of patients have experienced at least one adverse event when transitioning from hospital to home.^2^, ^3^ In Australia, medication‐related hospital admissions cost approximately $1.2 billion annually.^4^ Thus, health‐care providers have recognized the importance of improving medication safety during the transition from hospital to home.^5^, ^6^

Medication safety refers to the collective systems and strategies used to ensure medicines are used to reduce the risk of avoidable harm and enhance health outcomes.^7^ Factors such as age and polypharmacy (taking five or more regular medications), health status and health literacy are known to be associated with a higher risk of experiencing medication‐related problems.^8^, ^9^, ^10^ In the current literature, it has been suggested that the majority of issues related to medication safety are due to disorganized processes and designs within the health‐care system.^11^, ^12^, ^13^ Recently, research has predominantly focused on medication safety interventions that improve systems of care and professional behaviour.^14^ However, these were based on the perspective and assessment of the health providers and do not take into account patient perspective and experience.^15^, ^16^, ^17^

Patient engagement is widely advocated in addressing the issue of medication safety^17^, ^18^; however, its execution in practice is limited. Patient engagement is defined as the level to which: (i) patients are involved in their own health‐care and treatment,^18^ (ii) information flows between the patient and the health‐care provider^19^ and (iii) perceived personal control the patient has over their health status and well‐being.^20^ Few studies have investigated how empowering patients results in improving medication safety.^21^, ^22^, ^23^ Yet, these studies were limited by the fact that patients were simply provided information on how to self‐medicate, with no opportunity for discussion between health‐care professionals and the patient.

Patients desire to be involved in their health and participate in decisions about treatment.^24^ Recently, health‐care delivery has placed an emphasis on patient‐centred care. There is good evidence that this can lead to improvements in medication safety and patient satisfaction.^17^, ^25^, ^26^, ^27^ Nonetheless, an extensive review of the literature has shown that there are very few studies,^28^, ^29^ which have focused on the patients’ perspectives of medication safety post‐discharge, and to the authors’ knowledge there is no published Australian literature.

In this study, a series of open‐ended questions were included to gain a deeper insight and understanding of the patient's perspective on their attitudes, beliefs and preferences.^30^ The current literature in this area quantitatively identifies the types of medication‐related problems post‐discharge and interventions used to primarily reduce them, which are mainly orientated around the perspective of health‐care professionals. There is a need for health‐care providers to be informed about patients’ perspectives and experience of the health system. The aim of this study was to gather data on the patient's perspectives and experiences regarding MRPs following discharge from hospital. The broader aim of this project was to gather patient insights and experiences to eventually develop and investigate multidisciplinary integrative strategies that will support patients in safely navigating the health system and avoiding medication‐related problems.

## 2 METHOD

### 2.1 Study design

A self‐report online survey was designed. Survey participants were recruited through The Digital Edge, an online market research company. The Digital Edge has over 100 000 Australians registered with their online panels and is Gold accredited with the Australian Market Research and Social Organisations. A total of 68 883 potential participants received an email invitation to volunteer for the survey during September 2014. Those who agreed to participate were assessed for their eligibility to join the study using screening questions. Participants were included if: they were aged 50 years or older; taking five or more prescription medications; had been admitted to hospital with a minimum stay of 24 hours; and were admitted to hospital within the last 4 months and discharged from hospital within the last 1 month, to reduce recall bias. Participants were excluded if they were discharged to aged care or residential facilities. Eligible participants read the following in order to give the require participant information and gave consent before starting the survey: “Potential participants (registered with The Digital Edge) will be required to read the Participant Information statement, and tick a box below stating that they have read the Participant Information statement and agree to participate in the study prior to the questionnaire being completed. I have read the Participant Information statement and agree to participate in the study prior to the questionnaire being completed.”

Nine open‐ended questions in the survey were designed to elicit detailed information from participants from their viewpoint (Appendix). Participants were asked about their experiences of MRPs, their involvement in managing their medicines and the role of their health professionals during the discharge from hospital. The remainder of the survey questions was of a quantitative nature, the results of which are reported elsewhere.^10^

The study was approved by the Human Research Ethics Committee at the University of Sydney (Project number: 2014/569).

### 2.2 Data analysis

Patients’ responses to the open‐ended questions were coded, patterns in the data were then explored and thematically analysed using NVivo version 10.0 (QSR International, Doncaster, Australia). The development and refinement of codes and emerging themes were undertaken sequentially. The data were initially coded and interpreted by DE. The coded data and emergent themes were independently reviewed by other members of the research team. The data were organized into overarching concepts, and these were elucidated through the presentation of themes and illustrative quotes. Constant comparison enabled the identification of emerging themes within the concepts. Any disagreements in coding and interpretation were resolved through team discussion. The results presented outline the concepts and themes illustrated with verbatim quotations.

## 3 RESULTS

### 3.1 Participants’ characteristics

Five hundred and six participants completed the survey. Those who completed the survey ranged in age from 50 to 87 years (mean 64.0±8.2 years). The majority of participants were females (n=311, 61.5%) and identified their ethnic background as European/Caucasian (n=472, 93.3%). Fifty‐five per cent (n=278) of participants reported that they reside in metropolitan areas and 45% (n=228) in rural areas. Education attainment was equally spread between high school, technical/trade and university with 36.6% (n=185), 31.2% (n=158) and 32.2% (n=163), respectively. Thirty‐four per cent (n=174) of participants reported experiencing a MRP within the 1‐4 months after discharge from hospital.

### 3.2 Thematic analysis

The content of the open‐ended questions in the survey revealed two overarching concepts: *types of medication‐related problems and patient experiences* and *patient engagement in medication management*.

#### 3.2.1 Types of medication‐related problems and patient experiences

Three themes emerged from this concept: unwanted effects from their medicines, confusion about medications and unrecognized medications.

##### Unwanted effects from their medicines

The majority of MRPs reported were either (i) side‐effects of new medications or due to a dose increase or (ii) new medications that were not explained to them.

Started insulin without proper directions given also change to pills only found out after getting home (66‐year‐old, male)

I developed swollen breast as the result of taking Duodart (dutasteride and tamsulosin) … (69‐year‐old, male)

…Was accidently prescribed double the required dose of a certain medication (75‐year‐old, male)

[I] was given a medicine for restless legs and I was violently ill after taking it (73‐year‐old, female)

As a result, when asked how they felt about the problem(s), most reported that they were felt “worried”, “anxious” and/or “frustrated”:

Hallucinations while taking Endone (oxycodone) …felt very uncomfortable and out of control (62‐year‐old, male)

…I was worried I had breast cancer. I went to see the GP at the local medical clinic. The GP referred me to a cancer specialist who concluded that I did not have cancer (69‐year‐ old, male)

I had bad episodes of peripheral oedema (ankles and feet). The cause of this was said to be from a calcium channel blocker. [I] was also prescribed phentermine for weight loss but this caused significant tachycardia and unusually increased mood levels due to its amphetamine similarities. [This]…caused significant anxiety and frustration. (59‐year‐old, male)

Some participants reported that these unwanted effects were due to “changes in their medications” and lack of information given to them post‐discharge.

The hospital did not supply a medication list of what times to take the medications…I ended up with an adverse reaction due to taking medications too close together (56‐year‐old, male).

However, generally participants were satisfied with the service they received in primary care and thought that “nothing” could be improved in regards to the help they received from their health‐care professionals about their medicines during this transition from hospital to home.

##### Confusion about medications

The majority of participants reported that they felt confused about the changes in their medications upon discharge from hospital. This included: possible side‐effects and being informed on new or ceased medications.

Some of the medications that was taking before going to the hospital were stopped without any reason… [I felt] confused (62‐year‐old, male)

One of my usual medications that I have been taking for many many years, was not listed on my discharge medication sheet, and no‐one had said that I was to discontinue it, and there was also a new medication listed that I had no knowledge of, what it was for, or was taking while in hospital (63‐year‐old, female)

##### Unrecognized medication

Another theme that emerged from the data related to participants being given different medications. Most participants reported being given a different medication which included being given generic brands, the wrong medication, or someone else's medication:

…Tramal (which are green and yellow) they substitute with Tramadol (an alternative brand)…which is cheap and nasty and is white and two yellow stripes and as a pain killer about as good as aspirin. (60‐year‐old, male)

[I] started insulin without proper directions given also [there was a] change to pills… [I] only found out after getting home… [I felt] angry. (66‐year‐old, male),

My webster pack (dose administration aid) had different medications and some were left out. Also was discharge with some medication that was for someone else…[I felt] a little bit stressed… (78‐ year‐old, female).

#### 3.2.2 Patient engagement and medication management

Within this concept four themes emerged from the responses: informing patients, patient engagement, communication amongst health‐care professionals and conflicting advice.

##### Informing patients

Overall, participants felt that they would like health‐care professionals to give them more information on their new medications, such as the reasons for their use and side‐effects.

[I'm] not very happy since it (swollen breasts) was a side effect of taking Duodart (dutasteride and tamsulosin) and the doctor didn't tell me about the side effect. (69‐year‐old, male)

It would have been useful if someone had talked to me about the medications, how much and when to take them. (62‐year‐old, female)

… (The help I received could have been) improved by a health professional going through the list with me, and if there are any new medications, explaining what they are for etc. and if there are changes from normal medications, have that explained too (63‐year‐old, female)

##### Patient engagement

Interestingly, divergent views emerged when asked about the role of the patient in managing their medications. On the one hand, some participants believed it is the role of their health‐care professionals to make the decisions.

I trust my doctor so he/she should make those [medications] decisions for me (68‐year‐old, female),

…I am not a doctor and believe that they know/understand my conditions better than me. (77‐year‐old, male).

On the other hand, others believed it is a collaborative process.

The consumer should have the right to have some input into the medications they are prescribed. In my case my doctor prescribed a certain strength blood pressure medication that caused bad headaches for me… we worked out a compromise of taking a lower dose one day and the stronger one on the alternative day… (67‐year‐old, female),

It should be collaborative; patients need to know the reason for each medication without being bogged down by too much information. They also need to know that each medication they are prescribed is necessary and the best possible option for their particular condition(s). (57‐year‐old, female),

I was consulted by the doctor about my medicines & my opinions were taken into consideration, but I can see that patients with mild Dementia or other mental issues would need firm leading (78‐year‐old, female).

##### Conflicting advice

Some participants felt that they had been given conflicting advice by their health‐care professionals, particularly for those under the care of specialists.

…Confused because the hospital is telling me one thing about my medication and my doctor is telling something different. (52‐year‐old, male),

… Problem is that one doctor say you need this medication and strength and a another will say no you can not take this as it will put a strain on your high blood pressure (52‐year‐old, male),

Some of my heart pills were stopped yet when I went back to my cardiologist he said that is incorrect and put me back on them. [He] said he would look into it with the hospital (63‐year‐old, male).

##### Communication amongst health‐care professionals

Participants with multiple health‐care professionals believed that there should be more collaboration and communication.

I have nine specialists. They need to swap notes! (54‐year‐old, female),

Hospitals have no idea what treatments other hospitals give you and no one communicates with each other. The other day I had to sign a release form from my GP just so he could get the medical records from two hospitals concerning the treatment I have been receiving… (54‐year‐old, male),

The pharmacist rang the specialist. The hospital didn't change my heart tablets so problem is still the same. So tired. (65‐year‐old, female).

## 4 DISCUSSION

The purpose of this study was to gain insight into Australian patients’ personal experiences and perceptions during the transition from hospital to home. To the authors’ knowledge, this study has provided the first insights into patients’ experiences and perceptions of MRPs following discharge from hospital. Not only does this study acknowledge that this is still an on‐going issue in the context of the Australian health‐care setting, but it also highlights the continued need for (i) better communication amongst health‐care providers and (ii) that health‐care professionals should assess their patients’ preference for level of involvement in their health. It also presents evidence on conflicting views of patients regarding their desired level of involvement in their own medication management.

Confusion about medications was a powerful finding and at times were reflected in terms of feelings such as anger and anxiety. The results offered insights regarding (i) the changes in medication and (ii) the lack of information given to patients before discharge from hospital. According to Cua et al.^31^ patients are at high risk of MRPs when multiple medication changes occur around time of transition from hospital. Confusion may be associated with patients being given different brand of their medication, such as a generic product. A study by Forster et al.^32^ suggested that preparing patients in anticipation of discharge may help reduce MRP. In addition, a study by Cawthon et al.,^33^ reported that pharmacist‐based interventions such as giving a medication list before discharge from hospital and a follow‐up phone call were considered helpful and empowering. This suggests that a HCP intervention, to adequately inform patients about planned medication changes, could reduce the negative emotions (eg confusion and anxiety) associated with their transition from hospital to home.

According to Coulter,^24^ consumers desire to be actively engaged in their health and participate in decisions about treatment. Similarly, a study by Grimmer et al.^34^ identified that patients wanted to be more involved in discharge planning to ensure that they are able to address any practical situations they would face upon returning home. However, our data suggest that the views about roles that patients could take in managing their own medicines varied, from completely trusting in the health‐care professionals, to believing it should be a collaborative process.

Many factors may influence patients’ attitude towards their level of involvement. Charles et al.^35^ reported that most patients wanted some form of involvement in decision making; however, many felt that they did not have the required medical knowledge. Other studies have suggested that patients may prefer different roles depending on their health at different stages of their illness.^36^ This highlights the importance of HCPs being more sensitive to the needs of patients’ personal preferences and to provide patient‐centred care. It has been suggested whether patients feel empowered they may experience the benefits that are associated with involvement.^37^, ^38^, ^39^, ^40^, ^41^

The majority of participants reported wanting to be informed about their medication, and specifically about changes made to their medication list and possible side‐effects. An overseas study has examined the experiences of older patients and their carers, with regard to medication support post‐discharge. In that study, it was suggested that the lack of clear information about medication can lead to incorrect use of medicines at home, as well as confusion and anxiety.^28^ In addition, this study described participants wanting better communication amongst their health‐care providers. According to Kripalani et al.^42^ and Forster et al.^2^ most MRPs during the transition of care result from a breakdown in communication between prescribers.

Research has shown that good communication between health‐care professionals, regardless of the health‐care settings, reduce medication‐related problems during the transition of care.^42^, ^43^ Our findings confirm this, and highlight the personal cost of experiencing MRPs. Our study also demonstrated the importance of identifying a patient's desire to be engaged in the medication safety process. Despite a growing body of research focusing on interventions to improve patient participation during the transition of care,^6^, ^44^, ^45^ there is still a strong basis for future research to take account of the variation in patient's desire to be involved in their care. Our findings suggest that some people do not wish to be an active participant in this process.

### 4.1 Limitations

There are several limitations to acknowledge. First, the study sample did not reflect ethnic diversity in the Australian population, thus our results may not be generalisable to all ethnic groups. Second, people without access to the Internet could not participate. However, more than three quarters of all households in Australia have access to Internet,^46^ and participants may be more willing to share their experiences because they are not directly disclosing this in an interview, and they could answer questions at their own pace. Third, whilst the use of a survey has enabled data collection from a cross‐section of the population, the use of in‐depth qualitative interviews in this group of participants would have enabled a deeper exploration of issues. Last, this survey required participants to recall their experiences thus responses may have been influenced by memory bias.

### 4.2 Recommendations for practice

The content analysis suggests that (i) the level patients are willing to participate in decisions varies and (ii) more communication amongst health‐care professionals from different settings are required. Patient medication safety during the transition of care could be improved by changes within the health‐care system. Such modifications could focus on assessing these risk factors before patients transition from hospital to home, for example, asking questions addressing each risk factor as a checkpoint, identifying any barriers patients may have and acting as a tool to minimize any potential risk of experiencing MRPs before transitioning from hospital to home.

## 5 CONCLUSION

Patients have the potential to play an important role in their health‐care and medication safety. To the authors’ knowledge, this study is the first to report on the patients’ perspective of medication management during the transition from hospital to home in Australia. The study has revealed there are different perspectives on the level patients are willing to participate in decisions. The findings of this study can also be used to (i) develop questions to potentially reduce medication‐related problems, before patients are discharged from hospital, and (ii) conduct a pilot intervention for the feasibility of these questions. It is therefore important that further research is required in finding ways to design interventions integrating the patients’ perspective and developing ways to empower patients to ensure a smooth transition from hospital to home.

## CONFLICT OF INTEREST

The authors report no conflict of interest.

## APPENDIX 1 Open‐ended survey questions

### Medicine‐related problems you have had since your most recent discharge from hospital, and how these problems were addressed

1. Please describe the problem(s) you had with your medicines—what happened and when.
2. In what ways did the problem(s) affect you?
3. How did you feel about the problem (s)?
4. Why do you think the problem(s) occurred?
5. What did you do to try to resolve the problem? (For example, contact health care professions like your GP, nurse or pharmacist, family, friends, go to the internet, stop taking medicines)
6. How did they resolve the problem(s)?
7. In your view, what could have been done or how could the problem(s) be avoided in the future?

### Your medicines and role of your health professionals upon discharge from hospital

1. What, if anything, could be improved about the help you received from health professionals about your medicines during your transition of care from hospital to home?
2. What are your opinions about the role of the consumer/patient in managing medicines? For example, should consumers play a role in deciding which medicines and strengths are prescribed? Should the doctor make all the decisions?
